# Surgical removal of an unusual huge solitary fibrous tumor in the mediastinum: a case report

**DOI:** 10.1186/s13019-023-02366-3

**Published:** 2023-09-23

**Authors:** Parviz Mardani, Mohammad Nekooeian, Saba Zangeneh, Hooman Kamran, Reza Shahriarirad, Mohammad Hossein Anbardar, Armin Amirian, Masoud Vafabin

**Affiliations:** 1 Thoracic and Vascular Surgery Research Center, Shiraz University of Medical Sciences, Shiraz, 71936-13311 Iran; 2 Department of Surgery, Shiraz University of Medical Sciences, Shiraz, Iran; 3 School of Medicine, Shiraz University of Medical Sciences, Shiraz, Iran; 4 Health and System Research Center, Shiraz University of Medical Sciences, Shiraz, Iran; 5 School of Medicine, Fasa University of Medical Sciences, Shiraz, Iran; 6 Department of Pathology, School of Medicine, Namazee Teaching Hospital, Shiraz University of Medical Sciences, Shiraz, Iran

**Keywords:** Solitary fibrous tumors, Mediastinal neoplasms, Surgery

## Abstract

**Background:**

Intrathoracic Solitary Fibrous Tumors (SFT) mainly arise from the pleura; however, these tumors may also originate from the mediastinum. We present a rare case of posterior SFT extending to several mediastinal sites and with an unusual large size, successfully treated with surgical resection.

**Case presentation:**

A 66-year-old female presented with an initial manifestation of ambiguous pain in the chest and dysphagia and later developed pitting edema in both lower extremities and cachexia five months before admission. Chest imaging confirmed a mediastinal mass (17 × 15 × 8 cm) which was surgically removed. Immunohistochemistry confirmed the diagnosis of a solitary fibrous tumor with positive B-cell lymphoma 2, STAT6, and CD99, negative S100 and smooth muscle actin, and low levels of Ki67 (5–7%). The patient’s follow-up course was unremarkable.

**Conclusion:**

Mediastinal SFTs may grow extremely huge, with the potential to invade multiple adjacent sites. Surgical removal of the tumor remains the mainstay of treatment in these cases.

## Introduction

Solitary Fibrous Tumors (SFT), is a rare tumor originating from mesenchymal tissue. It is distributed equally between men and women, with a propensity to present in the fifth and sixth decade of life [1–3]. According to the present World Health Organization (WHO) classification of tumors, SFTs are now categorized as soft tissue neoplasms of fibroblastic or myofibroblastic origin that may originate anywhere in the body [4]. SFTs, mostly spindled to ovoid tumor cells with minimal cytoplasm lying in a patternless collagenous stroma, may be accompanied by a hemipericytoma-like condition characterized by a prominent vasculature [5].

SFTs make up fewer than 2% of all soft tissue masses and are often seen in the pleura; however, these tumors may also be detected in other areas of the body, including skin, nervous system, soft tissue, liver, lung, kidney, and thyroid [6–8]. Although most mediastinal tumors comprise thymoma, teratoma, thyroidal disease, lymphoma, congenital cysts, and neurogenic tumors [9, 10], SFT has been rarely reported in this location [11]. In addition to the unusual site of occurrence, these tumors are capable of growing to larger sizes compared to the average dimensions reported for mediastinal solitary fibrous tumors in literature [12]. Here, we present a case with huge posterior mediastinal SFT extending to several mediastinal sites, which is quite rare.

## Case presentation

A 66-year-old female presented with an initial manifestation of ambiguous pain in the chest and dysphagia and later developed pitting edema in both lower extremities and cachexia five months before admission. Past medical history was unremarkable, and the patient denied exposure to asbestos or other drugs or harmful substances. In the physical examination, vital signs were stable, and nothing significant was found. However, laboratory findings showed hypoalbuminemia (albumin: 1.9 g/dl).

**Fig. 1:**
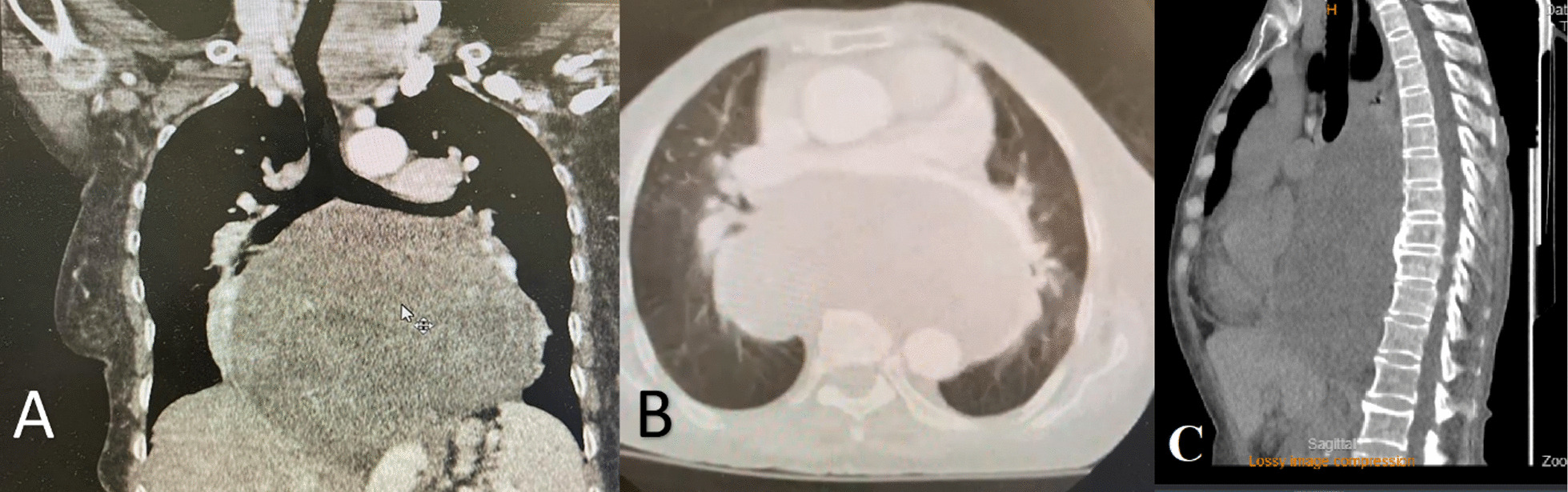
Chest computed tomography (CT) scan showing a large, hypodense, well-circumscribed posterior mediastinal mass (160*80 mm); **A** coronal view; **B** axial view; **C** Sagittal view

Before hospital admission, the patient underwent echocardiography, in which a mass was seen in the mediastinum. Therefore, a computed tomography (CT) was performed, showing an oval shaped, hypodense, well defined posterior mediastinal mass lesion measuring 160*80 mm in transverse and anteroposterior diameter, and 160mm in craniocaudal extension, causing anterior displacement of the heart, widening of subcarinal angle associated with mild to moderate right side pleural effusion. The adjacent thoracic vertebra was normal, however, linear atelectasis was also seen in basilar segment of right upper lobe (Fig. 1). Bronchoscopy was then performed, and a biopsy was taken. Pathological evaluation of the biopsy revealed a differential diagnosis of solitary fibrous tumor or fibromatosis. The patient was then referred to our center for tumor excision.

During her hospital course, chest ultrasonography revealed mild to moderate left-sided and moderate to severe right-sided pleural effusion. Therefore, a pleural drainage needle catheter (Pneumocath) was inserted. The patient also developed sudden tachycardia. The electrocardiogram displayed atrial fibrillation with a rapid ventricular response, which was treated with amiodarone (intravenous 150 mg over 10 min, followed by 360 mg over 6 h and 540 mg in the remaining 18 h).

After stabilization, the patient was scheduled for surgery. The thoracic cavity was entered from the fifth intercostal space (left posterolateral thoracotomy). Due to the large size of the tumor and the localization of the tumor and heart, sternotomy could not be performed. We attempted to approach the patient through the left posterolateral position and left thoracotomy. A huge posterior mediastinal mass was detected with extension to both right and left chest cavities with important adhesion to the pericardium, arch of the aorta and descending aorta, inferior vena cava (IVC), esophagus, inferior pulmonary vein, and lung tissue with 300 cc pleural effusion. We considered changing the position and entering the right cavity in case the tumor could not be removed with en bloc resection and left thoracotomy. After pneumonolysis, a meticulous dissection of the lesion was done, and the lesion was excised. An air leak test was done, the chest cavity was irrigated, and the operation ended after inserting two chest tubes. Fortunately, complete removal of the tumor was feasible through the left posterolateral position and right thoracotomy was not required.

**Fig. 2:**
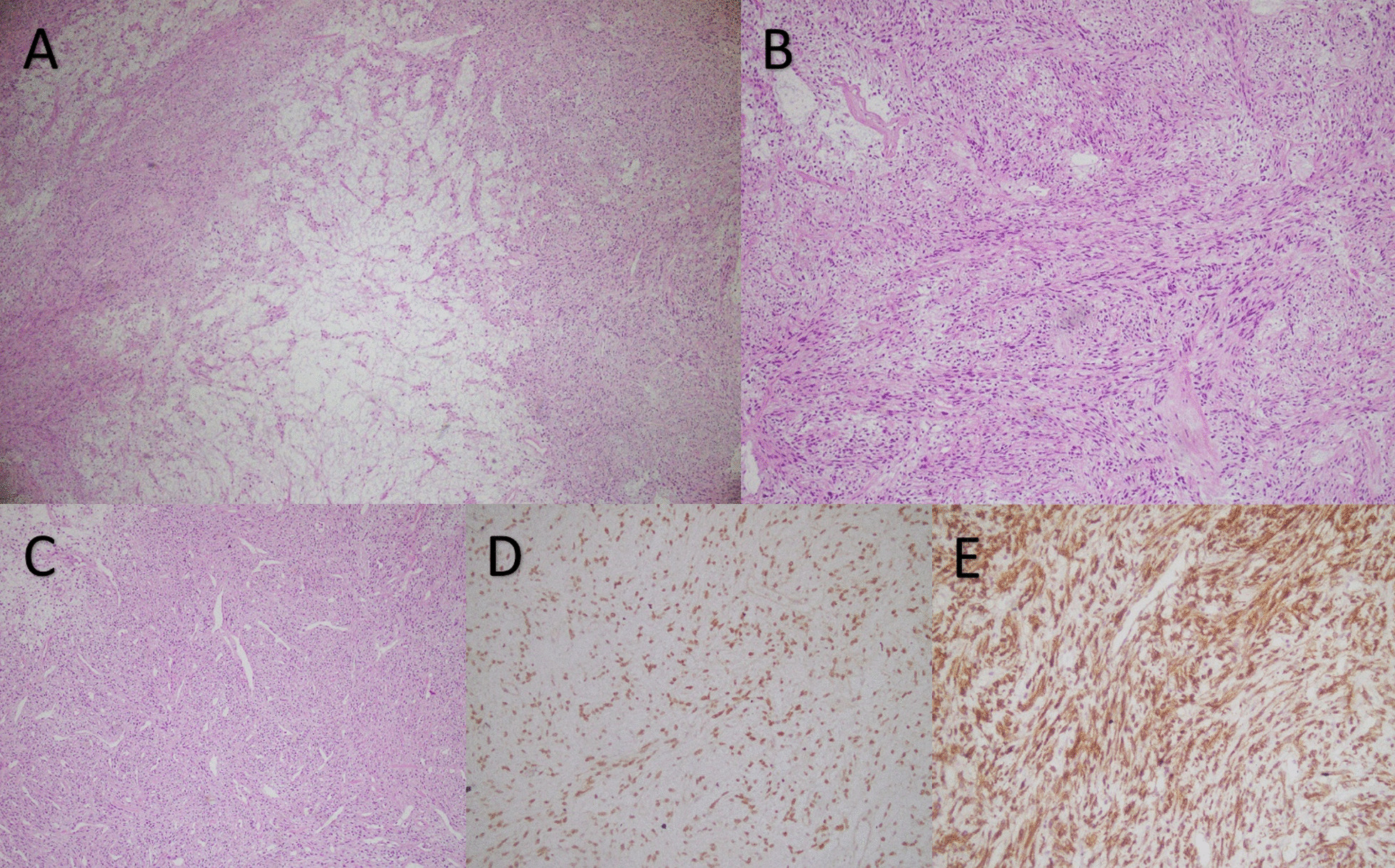
Histopathological evaluation; **A** the microscopic section shows a low power view of mass with hypocellular and hypercellular areas with myxoid degeneration (hematoxylin and eosin, ×40); **B** the microscopic section shows ovoid to fusiform spindle cells with indistinct cell borders arranged haphazardly in ill-defined fascicles (hematoxylin and eosin, ×100); **C** the microscopic section shows branching and hyalinized staghorn-like (hemangiopericytoma-like) vasculature (hematoxylin and eosin, ×100); **D** immunohistochemical reactivity for STAT6 (×200); **E**: immunohistochemical reactivity for MIC2 (×200)

A semi-ovoid lobulated creamy mass (17 × 15 × 8 cm) was received for histopathological examination. The capsular surface was bosselated, creamy, and vasculated. Cut sections showed homogenous creamy lobulated areas with fish flesh appearance and patchy necrotic areas. Necrosis was present at less than 5%, and one reactive lymph node was identified. Also, the mitosis rate was 1–2 per 10 high power field (HPF). Immunohistochemistry (IHC) confirmed the diagnosis of a solitary fibrous tumor with positive B-cell lymphoma 2 (BCL2), STAT6, CD34, and CD99 and negative S100 and smooth muscle actin (SMA). Furthermore, Ki67 levels were low (5–7%) (Fig. 2).

**Fig. 3:**
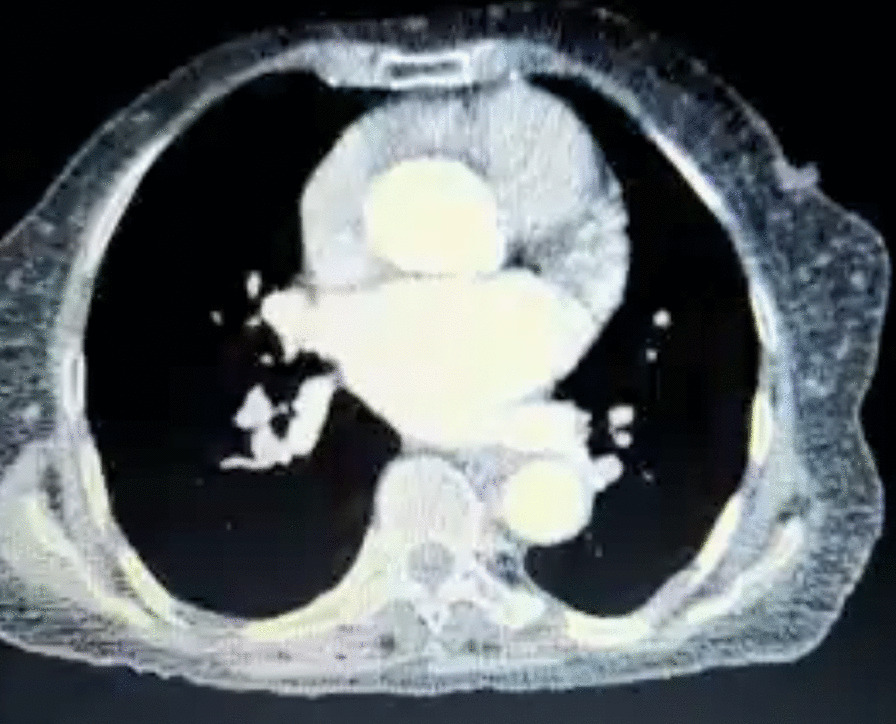
Postoperative computed tomography (CT) scan of a 66-year-old female, after left thoracotomy due to large solitary fibrous tumor of the mediastinum, demonstrating no significant findings

After the hospital discharge, the patient had pleural effusion, was malnourished due to poor feeding, and had hypoalbuminemia. However, during the follow-up, the feeding improved, albumin increased into the normal range, edema disappeared, and no complaints were reported after nine months of surgery (Fig. 3).

## Discussion

Intrathoracic SFTs mainly arise from the pleural; however, these tumors may also originate from the mediastinum. Although the anterior mediastinum is where most of the SFTs occur in the mediastinum [13], our patient presented with a huge posterior mediastinal mass with severe adhesions to the pericardium, arch of aorta, descending aorta, IVC, and esophagus. The majority of SFTs are asymptomatic and are found incidentally. Still, based on their size and location, they may present with a wide-ranging clinical manifestation ranging from dyspnea and chest pain [14, 15] to lower extremity edema resulting from compression over IVC [16]. Additionally, our patient had dysphagia and atrial fibrillation attributable to the sites under compression. Other cardiac electrical disturbances, such as right bundle branch block, have been previously reported [17], which signifies the importance of a thorough cardiac evaluation of such patients.

Although evaluating patients with signs and symptoms attributable to mediastinal masses usually begins with imaging modalities, including chest X-ray or CT scan, the definitive diagnosis is achieved by pathological examination. Depending on the presence or absence of external invasion, imaging characteristics of mediastinal SFTs on the CT scan vary from having sharp edges in case of no external invasion to unclear boundaries with surrounding organs when the invasion is evident. Moreover, in contrast to solid parts, which show enhancement, cystic necrotic components demonstrate no enhancement [12].

Although most SFTs follow a benign course, a feature like over four mitoses per 10 HPF, hypercellularity, presence of necrosis, and larger tumor size are suggestive of malignancy [2, 12, 18]. The mitosis rate identified from an isolated specimen of our patient was 1–2 per 10 HPF. However, necrosis was present (less than 5%), and the tumor was extremely huge, with dimensions of 17 × 15 × 8 cm. Moreover, the tumor had invaded multiple adjacent sites. Of note, a recent study reported the diameter of mediastinal SFTs between 3 and 17 cm [12].

Regarding the pathological characteristics of mediastinal SFTs, it might be challenging to differentiate them from other benign mesenchymal tumors [19]. Therefore, IHC staining methods are routinely employed to confirm the diagnosis. SFTs commonly express CD34, CD99, and BCL2 [20]. Additionally, strong nuclear STAT6 IHC staining, which can be used instead of costly molecular tests for detecting NAB2-STAT6 fusion genes, has been used to reliably differentiate SFT from other soft tissue tumors, tumors of the head and neck, gynecologic tract, and prostate[21]. As reported by Geramizadeh et al., the previously mentioned markers, if positive, were the most valuable in distinguishing SFTs, with CD34 and STAT6 at the top of the list [6]. In a confirming matter, the result of the IHC staining of our patients was positive for BCL-2, STAT6, and CD99. S100, an IHC marker that has been consistently reported to be negative in SFTs [22], was reported to be negative for our patient. However, Zhang et al. reported S100 to be unusually positive in a patient with mediastinal SFT [12]. Furthermore, the Ki67 index range of our patients was low (5%-7%), making the diagnosis of peripheral nerve sheath tumors (MPNSTs) unlikely [23]. SMA, a myogenic marker often present in a broad range of soft tissue cancers, including smooth muscle tumors, fibroblastic/myofibroblastic sarcomas, and angiomyolipomas, may be detectable in SFTs, but to a lesser degree [24].

Depending on several tumor characteristics, including size, location, and the presence or absence of external invasion, the therapeutic approaches chosen by surgeons are various. While VATS (video-assisted thoracoscopic surgery) is considered an acceptable method in smaller tumors with no external invasion, the preferred surgical method for large tumors close to mediastinal organs, especially those located in the posterior mediastinum, is thoracotomy [12, 17]. In our case, we entered the thoracic cavity via left posterolateral thoracotomy because of the tumor’s size and posterior location. In contrast to some previous instances in which the complete removal of the tumor mandates the surgeons to remove some parts of the pericardium or other adjacent structures [12], in this case, with thorough en-block resection of the tumor, we successfully removed the tumor without any need for concomitant pericardiotomy and no considerable insult to the surrounding structures. Furthermore, the indication of chemoradiotherapy in treating SFTs is still controversial [25], and such decisions are based on multidisciplinary consultations and the surgeon’s decision. Owing to the lack of distinctive criteria for forecasting the recurrence probability of surgically removed SFTs, all patients treated for SFTs must undergo periodic follow-up visits, regardless of the therapeutic or surgical strategy used to eradicate the tumors. Accordingly, the chest CT scan performed in the 9^th^ postoperative month was normal with no signs of recurrence or metastasis.

Regarding the role of chemotherapy in SFTs, we did not administer chemotherapy since the tumor was localized and was removed with resection with clear margins. Also, the patient had no signs of recurrence during her 9-month follow-up. Surgical excision with clear margins has been mentioned to be the treatment of choice based on feasibility and improved survival [26, 27]. Although some studies have supported the use of chemotherapy in the treatment of advanced SFTs, all data in this regard are related to single institutional and retrospective studies [28–30]. Park et al. reported the efficiency of conventional chemotherapy in stabilizing and controlling locally advanced and metastatic SFTs [28]. Among conventional chemotherapy agents, the temozolomide-bevacizumab combination and the sunitinib regimen are among the most commonly used and effective regimens for SFTs [31, 32]. On the other hand, some studies such as a report by Constantinidou et al. among patients with advanced SFTs, reported that palliative systematic therapy (anthracycline-chemotherapy) is of limited value (33). Therefore, further studies are required to explore the role of chemotherapy in this rare sarcoma subtype.

## Conclusion

Mediastinal SFTs may grow extremely huge, with the potential to invade multiple adjacent sites. Due to several reasons, the diagnosis and treatment of these tumors have remained complex. Firstly, they may present with wide-ranging clinical manifestations. Secondly, even with appropriate clinical suspicion, the radiologic features of mediastinal SFTs on chest CT-scan are not distinctive. Finally, the definitive diagnosis is only established with the help of pathologists and IHC staining methods. Surgical removal is the mainstay of treatment, however, even with the complete resection of the tumors, the latter may reoccur. Therefore, all patients should be closely monitored on a regular basis in order to detect the earliest signs of recurrence.

## Data Availability

All data regarding this case report has been reported in the manuscript. Please contact the corresponding author in case of requiring any further information.

## References

[1] Gold JS, Antonescu CR, Hajdu C, Ferrone CR, Hussain M, Lewis JJ (2002). Clinicopathologic correlates of solitary fibrous tumors. Cancer.

[2] Demicco EG, Park MS, Araujo DM, Fox PS, Bassett RL, Pollock RE (2012). Solitary fibrous tumor: a clinicopathological study of 110 cases and proposed risk assessment model. Mod Pathol.

[3] Cardillo G, Facciolo F, Cavazzana AO, Capece G, Gasparri R, Martelli M (2000). Localized (solitary) fibrous tumors of the pleura: an analysis of 55 patients. Ann Thorac Surg.

[4] Fletcher CD (2006). The evolving classification of soft tissue tumours: an update based on the new WHO classification. Histopathology.

[5] Brunnemann RB, Ro JY, Ordonez NG, Mooney J, El-Naggar AK, Ayala AG (1999). Extrapleural solitary fibrous tumor: a clinicopathologic study of 24 cases. Mod Pathol.

[6] Geramizadeh B, Marzban M, Churg A (2016). Role of immunohistochemistry in the diagnosis of solitary fibrous tumor, a review. Iran J Pathol.

[7] Davanzo B, Emerson RE, Lisy M, Koniaris LG, Kays JK (2018). Solitary fibrous tumor. Transl Gastroenterol Hepatol.

[8] You Y-H, Liu R-T, Zhang Y (2018). A large solitary fibrous tumour of the pleura: a case report and review of the literature. J Int Med Res.

[9] Tomiyama N, Honda O, Tsubamoto M, Inoue A, Sumikawa H, Kuriyama K (2009). Anterior mediastinal tumors: diagnostic accuracy of CT and MRI. Eur J Radiol.

[10] Duwe BV, Sterman DH, Musani AI (2005). Tumors of the mediastinum. Chest.

[11] Weidner N (1991). Intriguing case: solitary fibrous tumor of the mediastinum. Ultrastruct Pathol.

[12] Zhang L, Liu X, Li X, Tang Z, Shi C, Wang G (2017). Diagnosis and surgical treatment of mediastinal solitary fibrous tumor. Asia Pac J Clin Oncol.

[13] Witkin GB, Rosai J (1989). Solitary fibrous tumor of the mediastinum. A report of 14 cases. Am J Surg Pathol.

[14] Sung SH, Chang JW, Kim J, Lee KS, Han J, Park SI (2005). Solitary fibrous tumors of the pleura: surgical outcome and clinical course. Ann Thorac Surg.

[15] Baliga M, Flowers R, Heard K, Siddiqi A, Akhtar I (2007). Solitary fibrous tumor of the lung: a case report with a study of the aspiration biopsy, histopathology, immunohistochemistry, and autopsy findings. Diagn Cytopathol.

[16] Xue X, Chen J, Ma W, Zhu D, Zhang W, Chen G (2009). Mediastinal solitary fibrous tumor with right diaphragm invasion: report of a case. Surg Today.

[17] Suehisa H, Yamashita M, Komori E, Sawada S, Teramoto N (2010). Solitary fibrous tumor of the mediastinum. Gen Thorac Cardiovasc Surg.

[18] England DM, Hochholzer L, McCarthy MJ (1989). Localized benign and malignant fibrous tumors of the pleura. A clinicopathologic review of 223 cases. Am J Surg Pathol.

[19] Doyle LA, Vivero M, Fletcher CD, Mertens F, Hornick JL (2014). Nuclear expression of STAT6 distinguishes solitary fibrous tumor from histologic mimics. Mod Pathol.

[20] De Raet J, Sacre R, Hoorens A, Fletcher C, Lamote J (2008). Malignant giant solitary fibrous tumor of the mediastinum. J Thorac Oncol.

[21] Cheah AL, Billings SD, Goldblum JR, Carver P, Tanas MZ, Rubin BP (2014). STAT6 rabbit monoclonal antibody is a robust diagnostic tool for the distinction of solitary fibrous tumour from its mimics. Pathology.

[22] Erdag G, Qureshi HS, Patterson JW, Wick MR (2007). Solitary fibrous tumors of the skin: a clinicopathologic study of 10 cases and review of the literature. J Cutan Pathol.

[23] Kindblom LG, Ahlden M, Meis-Kindblom JM, Stenman G (1995). Immunohistochemical and molecular analysis of p53, MDM2, proliferating cell nuclear antigen and Ki67 in benign and malignant peripheral nerve sheath tumours. Virchows Arch.

[24] Al-Daraji W, Husain E, Zelger BG, Zelger B (2009). A practical and comprehensive immunohistochemical approach to the diagnosis of superficial soft tissue tumors. Int J Clin Exp Pathol.

[25] Chen L, Sang Y, Zhang Z, Yang W, Chen Y (2020). Strategy for initial en bloc resection of a giant mediastinal solitary fibrous tumor: Judicious usage of cardiopulmonary bypass. Thorac Cancer.

[26] Spitz FR, Bouvet M, Pisters PW, Pollock RE, Feig BW (1998). Hemangiopericytoma: a 20-year single-institution experience. Ann Surg Oncol.

[27] Espat NJ, Lewis JJ, Leung D, Woodruff JM, Antonescu CR, Shia J (2002). Conventional hemangiopericytoma: modern analysis of outcome. Cancer.

[28] Park MS, Ravi V, Conley A, Patel SR, Trent JC, Lev DC (2013). The role of chemotherapy in advanced solitary fibrous tumors: a retrospective analysis. Clin Sarcoma Res.

[29] Stacchiotti S, Libertini M, Negri T, Palassini E, Gronchi A, Fatigoni S (2013). Response to chemotherapy of solitary fibrous tumour: a retrospective study. Eur J Cancer.

[30] Cardillo G, Carbone L, Carleo F, Masala N, Graziano P, Bray A (2009). Solitary fibrous tumors of the pleura: an analysis of 110 patients treated in a single institution. Ann Thorac Surg.

[31] Park MS, Patel SR, Ludwig JA, Trent JC, Conrad CA, Lazar AJ (2011). Activity of temozolomide and bevacizumab in the treatment of locally advanced, recurrent, and metastatic hemangiopericytoma and malignant solitary fibrous tumor. Cancer.

[32] Stacchiotti S, Negri T, Palassini E, Conca E, Gronchi A, Morosi C (2010). Sunitinib malate and figitumumab in solitary fibrous tumor: patterns and molecular bases of tumor response. Mol Cancer Ther.

[33] Constantinidou A, Jones RL, Olmos D, Thway K, Fisher C, Al-Muderis O (2012). Conventional anthracycline-based chemotherapy has limited efficacy in solitary fibrous tumour. Acta Oncol.

